# Do frequency and frequency-related measures signal turn completion? An exploratory corpus study

**DOI:** 10.3389/fpsyg.2025.1610179

**Published:** 2025-12-04

**Authors:** Christoph Rühlemann

**Affiliations:** University of Freiburg, Freiburg, Germany

**Keywords:** word frequencies, turn-constructional unit, questions, storytelling, turn-transition

## Abstract

Speakers in conversation have access to word frequency information stored in the mental lexicon. This article examines whether word frequencies play a role as a turn-completion cue in conversation. Based on the Freiburg Multimodal Interaction Corpus (FreMIC), frequencies and frequency-related measures are compared in turn-constructional units (TCUs) from two types of action/turns that are systematically complementary with regard to turn transition: question TCUs, which exert pressure for the next speaker to take over, and storytelling TCUs, which largely resist transition. Based on these systematic tendencies, the focus is on question TCUs that result in speaker change and story TCUs that result in speaker continuation, thereby tying *turn-transition* inevitably to *social action.* We address two research questions: RQ #1 - *Do word frequencies in the TCUs follow an S-shaped pattern?* and RQ #2 - *Which frequency-related measures predict that a TCU will be followed by a turn transition or continuation?* To address RQ #1, a mixed effects model showed the same S-shape found in prior research in large corpora. To address RQ #2, a mixed-effects model was computed, with turn transition (TT) as a binary outcome variable. The model suggested that turn finality in question TCUs co-occurs with a more pronounced drop in word frequency toward the TCU end than in story TCUs. A follow-up analysis revealed a more asymmetrical (right-leaning) distribution of nouns in turn-final question TCUs. Information extracted from word frequencies may hence serve listeners in conversation as cues to anticipate turn completion in questions as opposed to turn continuation in stories.

## 1 Introduction

**Figure 1:**
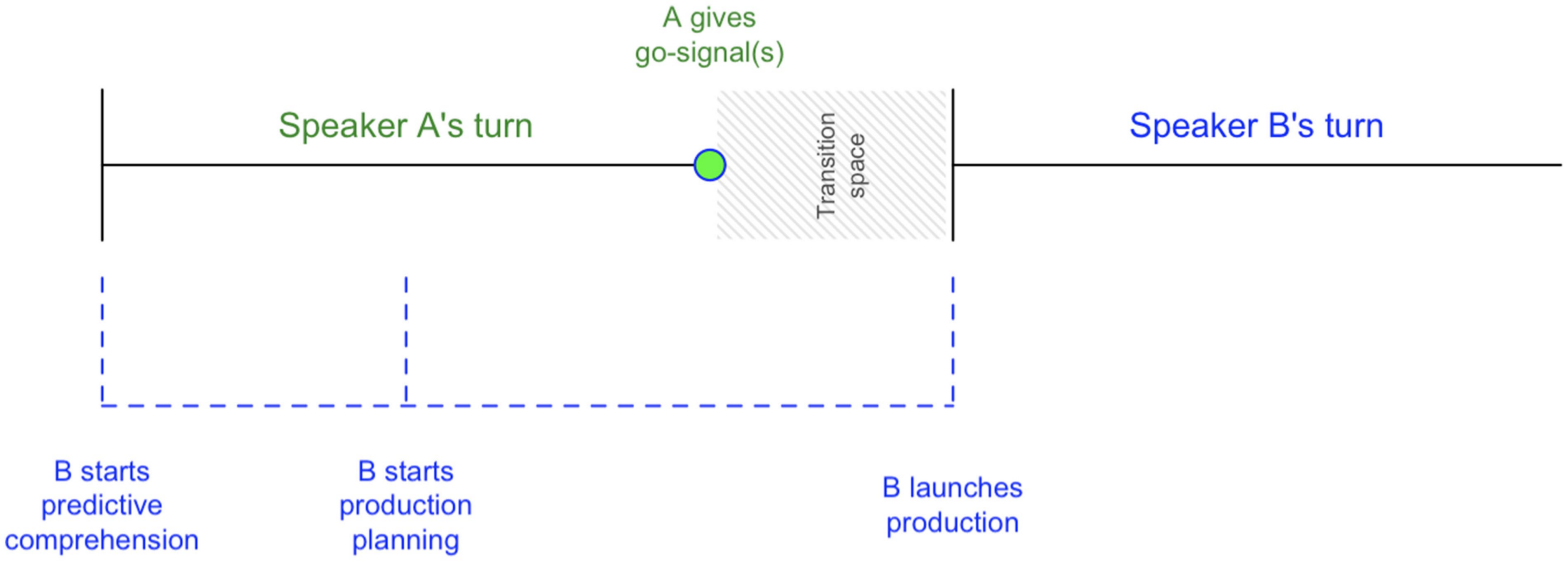
Schematic representation of the current consensus model on the synergy of early prediction and planning by the listener and late occurrence of *go*-signals that facilitate precision timing in turn transition.

Speakers in conversation across the world manage to produce a response to a prior turn with a small gap of around 200 ms (Stivers et al., 2009, p. 10588; Heldner and Edlund, 2010, p. 564). How is this precision-timing achieved? It is commonly assumed that listeners dual-task, predicting the unfolding action (speech act) and its time course while pre-planning their own response (Levinson and Torreira, 2015). The pre-planned response is launched as soon as the speaker gives the ultimate “go-signal” (Barthel et al., 2017: Holler and Levinson, 2019; Levinson and Torreira, 2015; Magyari et al., 2014; Gisladottir et al., 2018; Bögels and Torreira, 2015). The model is schematically depicted in Figure 1.^1^

Previous research on resources that listeners exploit in order to determine when a turn has, or is about to, come to a close has suggested a large number of such resources in all modalities. These resources do not only comprise “one–off“cues issued by the speaker upon turn completion, for example, a trail-off conjunctional or turn-final lengthening, but also include indexes derived from the turn as a whole that allow *long-distance projection,* such as lexico-syntactic predictability or rallentando (Levinson and Torreira, 2015, p. 13; Rühlemann and Gries, 2020; cf. also Sacks et al., 1974; Clayman, 2013; Magyari et al., 2014).^2^

In this study, we examine word frequency and related measures as another verbal resource to project and predict turn-completion. Frequency effects can be observed at almost any level of inquiry into language processing (Ellis, 2002). Our concern with frequency in this article is motivated by prior research suggesting an S-shaped distribution of word frequencies in conversational turns-at-talk (Yu et al., 2016; Klafka and Yurovsky, 2021; Rühlemann, 2020a, 2020b; Rühlemann and Barthel, 2024): frequencies start very high in turn-first position, then drop and level out until the last position in the turn, until they drop again steeply.

The S-shape pattern emerges very clearly and with little variation in the large conversational subcorpus of the British National Corpus (cf. Hoffmann et al., 2008) and is strong enough to also appear in the much smaller Freiburg Multimodal Interaction Corpus (FreMIC) (Rühlemann and Barthel, 2024).

Conversationalists are sensitive to word frequencies (Hasher and Chromiak, 1977; Hasher and Zacks, 1984). This transpires, for example, from the word frequency effect, that is, the fact that rare words are more slowly processed than common words (Oldfield and Wingfield, 1965; Jescheniak and Levelt, 1994; Indefrey and Levelt, 2004; Levelt et al., 1999; Johns et al., 2012). Given this sensitivity, the S-shaped distribution of word frequencies in turn would suggest the possibility that the drop in frequency in turn-last position represents a go-signal, that is, a one-off cue occurring upon turn completion, similar to an adress term (Sacks et al., 1974), or the return of the speaker’s gaze (e.g., Auer, 2018, 2021a, 2021b) However, given that frequencies decrease not just on the last word but overall within turns we wish to allow for the possibility that frequency serves as a resource for *advance-projecting turn completion* very much like syntax: just as syntax provides a structural envelope allowing the listener to predict the structural contour of the turn-in-progress, so frequency may provide a statistical envelope for the listener to predict the time course of the turn.

We thus hypothesize that the dynamic changes in frequency, including but not restricted to the drop in frequency on the turn-final word and the changes in frequency-related measures, do not go unnoticed by the listener and can be used by the listener as resources to (advance-)project (imminent) turn completion. As we have no access to recipients’ internal processes, to test the hypothesis, we investigate word frequencies and related measures in turns and their potential correlation with the actual occurrence or non-occurrence of turn transition observed in the sequence.

Specifically, we address two research questions: RQ #1 - *Do word frequencies in the TCUs follow an S-shaped pattern?* and RQ #2 - *Which frequency-related measures predict that a TCU will be followed by turn transition or continuation?*

Crucially, RQ #2 is examined by comparing questions and stories. These kinds of turns/actions differ fundamentally: questions are short, they consist mostly of a single turn-constructional unit, and they exert maximal pressure on the listener to respond (Stivers and Rossano, 2010, p. 29). Stories, by contrast, are extended turns, consisting of multiple TCUs, during most of which turn transition is avoided—typically until the climax, where assessments by the recipient are normatively relevant (Stivers, 2008). In (information-seeking) questions, the pressure is maximal: the provision of the sought information is normatively relevant; non-provision of the information may get negatively sanctioned (Stivers, 2013, p. 204). In Stivers (2010), for example, 93% of all questions were indeed followed by a turn transition. Storytellings provide a very stark contrast: they can be “very long stretches of talk being properly understood as being organized under the scope of a single sequence” (Schegloff, 2007, p. 215). They require the suspension of ordinary turn-taking (e.g., Jefferson, 1978) and entail a structural asymmetry, with the storyteller building up a succession of turn-constructional units (TCUs), and the listener filling the places between the units with recipient feedback in the form of vocal continuers (e.g.‘mm’, ‘uhu’, and ‘yeah’) (Goodwin, 1984) and/or visual continuers, such as nods (Stivers, 2008) and blinks (Hömke et al., 2017). Obviously, question turns can also be built out of multiple TCUs, and storytellings also come to a point where more action than issuing a continuer is expected from the interlocutor (namely at the story’s climax; cf. Stivers, 2008) and where, then, turn transition does occur. In addressing RQ #2, we therefore focus entirely on question TCUs that result in turn transfer and on story TCUs that do not lead to turn transition.

This methodological decision has important implications. The decision effectively means that turn transition is perfectly correlated with the type of action. Therefore, the present analysis does not claim to separate frequency-related features of transition *per se* from those associated with the social action of asking a question versus telling a story. Instead, what the study aims to identify are candidate frequency-related features that *co-occur* with transition-likely actions (questions) versus transition-resistant actions (stories). So, while we are using predictive modeling, prediction is used as a means to *discriminate* frequency-related features associated with turn-final question TCUs and, respectively, turn-medial story TCUs.

## 2 Data

### 2.1 The Freiburg multimodal interaction corpus

The data underlying the analyses in this article are part of the Freiburg Multimodal Interaction Corpus (FreMIC). Although small, FreMIC holds information of a breadth and level of detail not commonly seen in linguistic corpora (for a full description, see Rühlemann and Ptak, 2023).

FreMIC comprises ~30 h of video-recordings in 38 files transcribed and annotated in detail and featuring large streams of automatically generated multimodal data (e.g., eye gaze and pupil size). FreMIC’s total word count is 375,637. All conversations were annotated and transcribed in ELAN (Wittenburg et al., 2006). Two types of transcriptions were used: orthographic and conversation-analytic (e.g., Jefferson, 2004); the latter renders verbal content and interactionally relevant details of sequencing (e.g., overlap and latching), temporal aspects (pauses and acceleration/deceleration), phonological aspects (e.g., intensity, pitch, stretching, truncation and voice quality), and laughter. The underlying unit of analysis for transcription was the interpausal unit (IPU); that is, whenever a speaker stopped speaking for longer than 180 ms a new annotation was begun, a threshold that reflects the human 120 to 200 ms threshold for the detection of acoustic silence (Heldner, 2011; Walker and Trimboli, 1982; cf. also Levinson and Torreira, 2015 and Roberts et al., 2015, who also work with IPUs).

### 2.2 Participants

Forty-one individual participants were recruited to contribute to one or more of the 38 recorded conversations (total run time 30 h). Recordings lasted between 30 and 98 min (mean = 46.75 min, SD = 13.80).

The participants were explicitly told they were free to talk about anything that came to their minds. They were mainly students at Albert-Ludwigs-University Freiburg, as well as their friends and relatives [17 men, 21 women, 3 diverse/NA; mean age = 26 years (SD = 5.7 years)]. Most participants’ first language was English (*n* = 38, out of 41). All participants had normal or corrected-to-normal vision and hearing. Participants gave their informed consent about the use of the recorded data, stating their individual choices as to which of their data can be used and for what specific purposes. They received a compensation of €15 per hour for their participation.

### 2.3 The c7 tag set

All orthographic transcripts in FreMIC were part-of-speech tagged using the CLAWS web tagger (Garside and Smith, 1997) and its *c7* tag set.^3^ The *c7* tag set is a fine-grained tag set providing a total of 138 PoS categories (cf. Supplementary Material 1). The major advantage of such a fine-grained set is that it helps distinguish distinct morpho-syntactic functions of one and the same word form. For example, the word form *that* in English can take on a number of functions in context, for example, as a demonstrative as in *when was that?*, where in *c7* it is tagged *that_DD1*, a relativizer as in *the day that follows Christmas* (*that_CST*), a complex subordinating conjunction as in *now that you talk it’s fine* (*that_CS22*), and an adverb as in *it’s not that far* (*that_RG*).^4^ The accuracy rate for the *c7* tagset is 96–97% (Rayson, personal email communication; cf. also Leech et al., 1994; Garside and Smith, 1997).

### 2.4 The data subsets

#### 2.4.1 Data selection

Question turns can be used to do a wide range of things, such as initiating repair, confirming, and assessing (e.g., Stivers, 2010). This study focuses on information-seeking questions.^5^ Four syntactic types were targeted: *wh*-questions, polar questions, declarative questions, and multi-clausal *or*-questions, such as *is it!mult!iple singers for the band or is she like the main one °then° =*.

The stories for this analysis were selected from the data used for a prior analysis (Rühlemann and Trujillo, 2024) based on the condition that they be ‘big-package’ stories involving canonical story structure with (optional) story abstract, background, complicating events, and climax (Labov and Waletzky, 1967; Labov, 1972; cf. also Goodwin, 1984).

Both the questions and the storytellings selected were elaborately pre-processed in a joint effort by multiple researchers (Rühlemann et al., n.d.). The pre-processing is detailed in the following.

### 2.5 Data pre-processing

Turns can be single-unit turns or multi-unit turns (cf. Robinson et al., 2022). Storytellings are virtually always such extended turns stretching over multiple turn-constructional units (TCUs), and question turns can harbor a complex structure too. The questions and storytellings that form our data were therefore manually segmented into TCUs and whatever other units were found.

TCUs were operationalized as “coherent and self-contained utterance[s], recognizable in context as ‘possibly complete’” (Clayman, 2013, p. 151) so that another speaker could legitimately step in. “Completeness” was investigated in terms of syntax, prosody, and/or pragmatics (Clayman, 2013). While syntax served as the main guide for identifying TCU boundaries, prosody could override it in certain cases—specifically when (i) an extension, though grammatically complete, was bound to the prior unit through intonation, and (ii) the break between the core TCU and its extension was made audible by a shift in pitch or contour. The TCU segmentation in questions and stories is detailed in the following.

#### 2.5.1 TCU segmentation

##### 2.5.1.1 TCU segmentation in questions

Question *turns* can be single-TCU turns or multi-unit turns exhibiting a more complex structure due not only to the occurrence of more than one question-TCU but also to the speaker’s use of other, non-TCU or non-question material. As is generally the case (cf. Robinson et al., 2022), most questions in the data were single-TCU turns; question turns with two or more question-TCUs were less frequent. Consider extract (1), where the distinct turn components are separated by **|**:

(1) [F04, Sequ 35]
01 	B:	[but] =°but° w- if you say it's a Dachgeschoss top floor is it like 	02			(0.493) slanted? **| pol**
03		and can you actually [walk?] **| pol**

In multi-unit question turns, speakers often also use TCUs that do not perform the action of asking a question but that do other things (labeled *non-Q*), as in extract (2):

(2) [F01, Sequ 1]

01 	A:	>like I do n't understand< **| nonQ**
02		sorry **| nonQ**
03		like how old's your mom¿ **| wh**

The first TCU *> like I do n’t understand<* as well as the following TCU *sorry* are clearly not questions; only the third TCU *like how old’s your mom¿* serves to request information.

Question-TCUs are sometimes extended by a turn increment; to the extent that these were syntactically and/or prosodically separated from the preceding question TCU, they were treated as a separate, extension TCU (labeled *ext*), as shown in extract (3):

(3) [F07, Sequ 109]

01 	C:	<what would you call it> **| wh**
02		this **| frg**
03		you know when you don't clean your sink [like ever] **| ext**

Here, the first segment represents the question TCU; it is followed by the fragment *this*, and finally extended with *you know when you do not clean your sink [like ever].*

Not all verbal material a speaker uses in a turn may be part of a TCU; these components are referred to as fragments (labeled *frg*). They include syntactically incomplete utterances, turn-initial particles, as well as turn-final particles. Such particles are treated as fragments only if they are separated from the TCU by an intonation boundary (indicated in the transcripts by “,” “?” or “¿”). Contrarily, if they are intonationally integrated into the TCU, they are treated as part of the TCU. For example, in extract (4), the (repeated) particle *so* heading the question-TCU *[so] so do you just stay on the cruise ship*, is intonationally integrated into the TCU and therefore considered a part of it. By contrast, the trail-off conjunctional *o:r = following* the question-TCU is intonationally separated and therefore a fragment:

(4) [F08, Sequ 207]

01 	C:	[so] so do you just stay on the cruise ship, **| pol**
02		o::r= **| frg**

##### 2.5.1.2 TCU segmentation in storytellings

Storytellings are often considered multi-unit turns as they are of extended length and consist of several, often numerous TCUs. Storytellings are thus large “projects,” whose completion is potentially projected by a story preface adumbrating the story’s high point and/or the storyteller’s stance toward it (Stivers, 2008). Once the co-participants grant permission to carry out the telling project, they also implicitly agree to a suspension of ordinary turn-taking for the duration of the story, giving the storyteller the right to an extended turn, involving a series of narrative TCUs.

However, not all TCUs a storyteller uses in telling their story are *per se* a narrative TCU. Story recipients may insert comments or ask questions in mid-story position, which the storyteller responds to; alternatively, storytellers themselves may interrupt the telling, for example, to recruit story recipients in a word search. These actions/TCUs by the storyteller are not narrative TCUs with suspended turn-taking. Rather, in that the storyteller responds to or seeks to initiate a recipient’s action, these TCUs are interactive ones in which normal turn-taking is briefly resumed. Moreover, even in uninterrupted, smoothly delivered storytelling, turn transition is not avoided everywhere. On the contrary, based on a conceptualization of “storytelling as an activity that both takes a stance toward what is being reported and makes the taking of a stance by the recipient relevant” (Stivers, 2008, p. 32), the story climax can be considered the transition-relevance point in storytelling interaction. For it is here, at or around the story’s high point, that story recipients are expected to actively take a stance on the story events—a stance that, preferably, “mirrors” the storyteller’s. That is, those narrative TCUs that depict the story’s high point are then designed, not to avoid, but to initiate turn transfer.

To illustrate, in (5), (where narrative TCUs are labeled *narr* and interactive TCUs are labeled *int*), speaker A is telling a story about his father’s career as a diplomat, which the storyteller bills as a *sad story* (not shown in the transcript). The father’s career hit a bump when the US reached its *maximum budget deficit* (line 04). At this point in the telling, the storyteller changes into interactive mode by asking *°what’s°*(.) (line 05) *°what’s that called again?°* (line 06), to which none of the two recipients respond immediately, so he continues with the story *so there’s a government shutdown<* (line 08) before, finally, recipient A does proffer *the [fiscal cliff]* (line 09) as a candidate term. Speaker A immediately confirms this as the searched-for term by repeating it emphatically (line 10) and reaffirming it (line 11), and then resumes the telling (lines 13 and 15). In line 15, the telling reaches (the beginning of) the story climax: as a result of the fiscal cliff, the father’s position *as a diplomat is cut*—which is the “sad” event that the story set out to relate. As per preference structure (Stivers, 2008), recipient A answers empathically *wow*:

(5) [F16, story “Sad story”]

01	C:	=u:m and then they had a ↑budget↑ cut (.) **| narr**
02		um oh I mean u:h: **| frg**
03		the US reaches its um budget deficit, **| narr**
04		>its maximum budget deficit< **| narr**
05	**—>**	°what's° (.) **| frg**
06	**—>**	°what's that called again?° **| int**
07	**—>**	°governmental° tt >I don't know **| int**
08		so there's a government shutdown< **| narr**
09	A:	the [ fiscal   cliff  ]
10	C:	    [a:nd the !fiscal!] **| frg**
11	**—>**	yeah °ye[ah]° (.) **| int**
12		and u:h **| frg**
13		and so as a result all new positions are cut **| narr**
14	A:	[mm    ]
15	C:	[uh and] his position as a as a diplomat is cut **| narr**
16	A:	wow

Storytellers frequently, especially around story climaxes, use direct speech (or constructed dialog or enactments, which clusters around climaxes; cf. Labov, 1972; Li, 1986; Mathis and Yule, 1994; Mayes, 1990; Norrick, 2000; Clift and Holt, 2007; Rühlemann, 2013), as exemplified in extract (6); the content of direct speech (or, as in lines 01 and 06, silent gesture) is indicated by ~; TCUs containing direct speech are labeled *dr*:

(6) [F27, story “Black Forest”]

01	A:	I was like ~yo¿ ((imitates typing on keyboard))~ **| dr**
02		~YO GUYS I think I'm gonna go to this place called <!Frei!:bu:rg 		03		a:nd> there's !some!thing here called the Black !Fo!rest~**| dr**
04		and it's <almost like> everything STOPped **| narr**
05		like ~↑weow↑~ **| dr**
06		and everyone just stopped like ~((freezes/2.5))~ **| dr**
07	B:	[((laughs))]
08	A:	[it  got like] !NO! REACtion **| narr**

Another critical part of the data pre-processing was the annotation of *Turn Transition* (TT), the response variable in model #2, addressing RQ #2.

#### 2.5.2 Turn-transition coding

##### 2.5.2.1 Turn-transition coding in questions

The critical variable in this study, indeed the outcome variable of the model addressing RQ #2, is *Turn Transition* (TT), a binary variable recording whether a TCU led to a speaker change and turn transition or not. In single-TCU questions, the coding as such was obvious (except for the few cases where the first response was by the non-selected third participant; cf. Lerner, 2019). In complex question turns, TCU segmentation allowed us to identify the TCU that the speaker’s response was a response to:

(7) [F01, Sequ 5]

01 	C:	[what] type of:: tours is it **|  wh**
02		is it [(like    a long)] ti:me¿ **| pol**
03		[ or  ] **| frg**
04 	A:	      [it's cruise ship]
05		[tours]

In extract (7), speaker A’s response “*it’s cruise ship tours”* specifically responds to speaker C’s first question-TCU “*what type of: tours is it”* for two reasons: first, the response overlaps with key lexical elements of the second question-TCU *is it (like a long) ti:me¿,* and it is therefore unlikely that speaker C can even hear this question-TCU, let alone process it. Second, the response “*it’s cruise ship tours”* is both syntactically and semantically fitted to the *wh*-question “*what type of tours is it”* but not to the polar question *is it like a long time¿*, which would require a *yes/no*-type answer. In QA sequences such as these, the variable Turn Transition (TT) was coded “yes” only for the responded-to question-TCU; the TCU(s) to which the response was not fitted were coded “no.” In cases where the response was fitted syntactically and semantically to more than one TCU, the question’s *last* and fully audible question TCU was coded as the one leading to the turn transition.

In extract (8), for instance, the question turn is made up of a sequence of three question-TCUs (two declarative question-TCUs and one *or* question-TCU), all three syntactically aligned (i.e., answerable by *yes/no*), but only the last (*or*-)TCU is coded as the one leading to turn transfer:

(8) [F08, Sequ 167]

01 	A:	so it 's like not really like Fra:nce **|  decl**
02		it 's like a mix <°of the two°> **|  decl**
03		or is it like !real!ly French **|  or**
04		[like a r-] **|  frg**
05 	B:	[no   it’s ] it's I I guess it's a bit like (.) Alsace=

Two types of sequences were excluded from the analysis. Sequences such as (9), where a gap of more than 1 s ensued between the (final) question-TCU and the answer, were omitted from further analysis, as a gap of this length is far beyond the “regular” gap of around 200 ms, potentially indicating comprehension problems, a dispreferred answer, uncertainty as to who is selected as the next speaker, and so on. In extract (9), it appears that the gap of 1.19 s is a harbinger of a disaligned answer (an answer, in this case, whose truth value is compromised due to it being *individual* and *subjective* only):

(9) [F12, Sequ 226]

01 	B:	but how is it for you¿ **|  wh**
02		do you feel like <you: remember more than> fifty percent of what you 03		learned in your bachelor 's degree? **|  pol**
04		or **|  frg**
05		like what what would you say¿ **|  wh**
06	**—>**	(1.190)
07 	A: 	°so° !ob!viously this is very like
08 	B:	like it 's
09 	A:	[individual  (.) ye:ah  exactly  so  it's  very]
10 	B:	[very sub!ject!ive (depending on °how it goes°)]

Sequences as in extract (10), where the answer is referenced to a TCU-extension, were removed from the data set given their lack of syntactic and semantic independence from the preceding question-TCU (indeed, they cannot ‘survive’ without them):

(10) [F04, Sequ 50]

01	A:	u:h the guy (.) **| frg**
02		you remember Urick? **| pol**
03		and there was like a room directly 	across of me¿ **| ext**
04		that a guy moved out and his girlfriend?= **| ext**
05	B:	=°°yeah°°=

###### 2.5.2.2 Turn transition coding in stories

TCUs labeled *int* were coded as facilitating speaker change (Turn Transition = “yes”) regardless of their position in the story. By contrast, TCUs labeled *narr* and *dr* were both coded as avoiding speaker change (*Turn Transition* = “no”) only in pre-climax position; narrative TCUs at or around the story climax eliciting engaged recipient response, such as the one in line 15 in extract (9), were coded as inviting turn transition (*Turn Transition* = “yes”).

To ensure replicability, interrater-reliability (IRR) analyses were carried out both for TCU-segmentation and *Turn Transition* (TT) coding.

#### 2.5.3 Interrater-reliability analyses

##### 2.5.3.1 Interrater-reliability for TCU segmentation

From the 457 QA sequences, 92 sequences (20%) were randomly sampled, and the IPU transcriptions available in FreMIC were TCU-segmented by a second rater. The 13 stories were each divided into three same-size intervals (c. 33%), and one interval was randomly sampled from each story. The IPU transcriptions available in FreMIC for those intervals were TCU-segmented by a second rater.

The agreement percentage for question-TCUs in which both raters segmented exactly the same words was 83.58%, and the percentage for storytelling-TCUs with the exact same segments and hence the same words was 71.68%. This lower agreement rate likely reflects the fact that the IPUs underlying the segmentation in stories tend to be markedly longer than the IPUs underlying questions, thus allowing more divergent codings. This greater length of IPUs also transpires from the greater length of storytelling TCUs: as shown in Table 1 (cf. Section 2.5.6), the mean number of words in story TCUs is 7.20 (median = 6, SD = 4.58) as opposed to 6.04 in questions (median = 5, SD = 3.45) and the mean duration is 1,851 ms (median = 1,440 ms, SD = 1,506) as opposed to 1,450 ms in questions (median = 1,218, SD = 1,005).

**Table 1:** Descriptive statistics: Number of words (*N_w*) and durations of TCUs in original data (1,074 TCUs).

Type	N_w				Duration (ms)		
	Range	Mean	Median	SD	Median	Mean	SD
all	1–39	6.53	6	4.01	1,300	1,621	1258.34
question	1–33	6.04	5	3.45	1218.5	1450.43	1005.54
story	1–39	7.20	6	4.58	1,440	1851.35	1506.74

##### 2.5.3.2 Interrater-reliability for turn transition (TT)

In the questions subset, the IRR analysis for *Turn Transition* (TT) was carried out only on QA sequences with more than one question-TCU (coded *wh*, *pol*, *decl*, or *or*), as there is no choice as to which TCU is answered if there is just one. This subset consisted of 72 sequences; 24 of them (c. 33%) were rated by a second rater. In the storytellings subset, the narrative TCUs (coded *narr* or *dr*) as well as the interactive TCUs (coded *int*) were selected; a proportion of 33% of them were randomly sampled and coded for *Turn Transition* by a second rater.

The agreement percentage for *Turn Transition* coding in questions and storytellings taken together was 91.2%, yielding a Cohen’s Kappa of 0.706 (*p* < 0.001), which indicates substantial interrater agreement (cf. Landis and Koch, 1977).

#### 2.5.4 Statistical overview of the data

The analysis started out with a total of 1,074 TCUs. The descriptive statistics for this original data are shown in Table 1.

The mean number of words in the TCUs was 6.5, their mean duration 1,621 ms; for comparison, TCU mean length in Hömke et al. (2017) was 1,754 ms.

To address RQ #1 — *Do word frequencies in the TCUs follow an S-shaped pattern?*—TCUs with fewer than three words were excluded as no development of frequencies can be read-off of them; the number of thus-excluded TCUs was 100 (or 9.31% of the total 1,074 TCUs), leaving model #1 with 974 TCUs produced by 29 distinct participants. (For RQ #1, the distinction between question- and story-TCU was not relevant.)

Addressing RQ #2—*Which frequency-related measures predict that a TCU will be followed by turn transition or continuation?*—the data set was further reduced. Given the focus of RQ #2 on the (potential) effect of frequency-related measures on *Turn Transition*, question-TCUs that did not result in turn transition were excluded, thus keeping only question-TCUs coded “yes” on *Turn Transition* (TT), as were story-TCUs that did result in turn transition, thus keeping only story-TCUs coded “no” on *Turn Transition*. As noted, this decision intimately ties the results of the predictive modeling undertaken to address RQ #2 to the social-action type: whatever significant effects we may observe cannot be taken as features of turn transition in itself, independent of the type of social action in which it occurred, but will discriminate frequency-related features of turn transition in (i) transition-ready question TCUs and (ii) transition-resistant story TCUs.

After all reductions were made, model #2 was based on 876 TCUs. Of these, 457 were question-TCUs asked by 29 distinct participants and 419 story-TCUs occurring in 18 stories told by 13 participants (who were a subgroup of the 29 questioners). The participants’ demographic details are given in Table 2.

**Table 2:** Participants’ gender, age, and L1 (first language).

Gender	Age	L1
Male: 13	Range: 20–49	English only: 20
Female: 13	Mean: 26.5	English + other: 6
cis-Fe/Male: 2	Median: 26	not English: 2
*NA*: 1	SD: 6.42	*NA*: 1

#### 2.5.5 Computation of word frequencies

As noted, FreMIC’s total word token count is 375,637. A frequency list was computed for the whole corpus, based on *c7* word-tag combinations, giving the absolute word token frequencies for any *c7* word-tag combination. Frequencies were normalized per 1,000 words and log-transformed (to the base of 2). The top 10 most frequent *c7* word-tag combinations in FreMIC are shown in Table 3: as is to be expected from a conversational corpus, personal pronouns as well as interjections such as *yeah_UH* are ranked highly, whereas noun-related items such as *the_AT* and a*_AT1* are less highly-ranked than in general or written corpora (e.g., Biber et al., 1999; Stubbs, 2001; Rühlemann, 2007):

**Table 3:** Top 10 most highly-ranked c7 word-tag combinations in FreMIC.

w_c7	freq	f_norm	rank
I_PPIS1	16,448	43.7869539	1
it_PPH1	10,440	27.8460322	2
yeah_UH	10,270	27.4413862	3
and_CC	10,094	26.9009709	4
the_AT	9,660	25.8228023	5
you_PPY	8,583	22.9290512	6
‘s_VBZ	8,315	22.2368936	7
like_II	6,945	18.5418369	8
a_AT1	6,602	17.6100863	9
was_VBDZ	4,781	12.7782939	10

Assigning the corpus frequencies to the words in the TCUs presented a challenge because, as noted, in FreMIC, the underlying unit of observation is the IPU, and the *c7* word-tag ‘transcriptions’ available in FreMIC are for IPUs as well. Large numbers, however, of the TCUs obtained from manual segmentation in ELAN did not map onto these IPUs either because a TCU was just one part of an IPU or a TCU spanned two or more IPUs. Mapping *c7* word-tags and their frequencies to the words in the TCUs, therefore, required additional work.

To illustrate, the utterance *so wait (wha-) [when was this]* in excerpt (11.a) represented one uninterrupted IPU in FreMIC. It is associated with the string of *c7* word-tags shown in (11.b). During the TCU-segmentation process, the IPU was broken up into three segments, as shown in (11.c).

(11.a)
so wait (wha-) [when was this]

(11.b)
so_RR wait_VV0 wha-_UNC when_RRQ was_VBDZ this_DD1

(11.c) [F36, Sequ 574]

so wait **| nonQ**
(wha-)  **| frg**
[when was this] **| wh**

To map the c7 word-tags to each TCU segment, the c7 word-tag strings in (11.b) had to be separated into the exact same segments using a multi-step coding procedure in R so that the c7 word-tag segments could be matched to their corresponding TCU segments, as shown in (11.d):^6^

(11.d):

so wait **| nonQ** so_RR wait_VV0
(wha-)  **| frg**  wha-_UNC
[when was this] **| wh**  when_RRQ was_VBDZ this_DD1

The next pre-processing step was to assign to each *c7* word-tag in the TCU segments their total corpus frequencies.

#### 2.5.6 Computation of frequency-related measures

While there is some agreement that conversationalists constantly monitor relative word frequencies during conversation (Shapiro, 1969; Hasher and Chromiak, 1977; Hasher and Zacks, 1984), the question of *how* they do it is largely an open question.

It is, for example, unclear whether conversationalists monitor frequencies relative to the turn-so-far (i.e., the Saussurian *parole*) or the language as such (i.e., the Saussurian *langue*). If word frequencies are monitored relative to *langue*, the relative word frequencies are ‘simply’ retrieved from the mental lexicon in which they are stored (e.g., Jaeger, 2010; Seyfarth, 2014), to the extent that the corpus can be seen as a microcosm reflecting the macrocosm of *la langue*,^7^ this would suggest that speakers make use directly of corpus frequency values *independently of one another*. Consider, for example, the question-turn *What’s a mountain for you?.* As shown in Table 4, the lowest normalized frequency is for the noun *mountain*, a rather rare noun (and, in English, rarity is highly correlated with nouns; cf. Rühlemann and Barthel, 2024), whereas the highest frequencies are for the shortened form of the verb *is* and the pronoun *you.*^8^

**Table 4:** Log-transformed normalized rank and frequency values for *What’s a mountain for you?* [F01, Sequ 9].

Word token	*c7* word-tag	f_norm	f_norm_log
what	what_DDQ	5.5586	1.7153
‘s	‘s_VBZ	22.2369	3.1018
a	a_AT1	17.6101	2.8685
mountain	mountain_NN1	0.0426	−3.156
for	for_IF	5.3243	1.6723
you	you_PPY	22.9291	3.1324

If, by contrast, frequencies are monitored with reference to *parole*, that is, to their immediate context of use, the frequencies are still retrieved from the mental lexicon but are additionally put in relation to one another.

An established method to capture speakers’ monitoring of relative frequencies in turns/TCUs is *surprisal* (e.g., Piantadosi et al., 2011; Seyfarth, 2014). Surprisal may be part of the resources listeners deploy to predict the TCU’s lexico-syntactic path so as to be able to anticipate the TCU end and speed up their response (cf. Magyari et al., 2014: 2537; cf. also De Ruiter et al., 2006). To measure *surprisal*, the Conditional Probability of each word is calculated given the word or words preceding it; that probability then is converted to *surprisal* by taking the negative log of each probability.

We calculated *surprisal* based on bigrams, establishing how unexpected word B is given word A, C given B, D given C, and so forth. This method and the related unigram and trigram-based methods have some currency in linguistic research (e.g., Klafka and Yurovsky, 2021; Rühlemann and Gries, 2020; Trujillo and Holler, 2025); it implies that upon listening to a current speaker, conversationalists experience an increment to a turn-so-far (i.e., the next word) as more or less surprising based on a comparison of that increment’s frequency with the frequency of its combination with the immediately prior word(s).^9^

To illustrate, as shown in Table 5, in the question *What’s a mountain for you?*, it is to be expected that *surprisal* is highest on the word *mountain*, given that the indefinite article preceding it is highly common, whereas the noun is rare.

**Table 5:** Bigrams, Surprisal, Cumulative ngram, (log-transformed) Cumulative Ngram Frequency (CNF) for *What’s a mountain for you?* [F01, Sequ 9].

Bigram	Surprisal	Cumulative ngram	Cumulative Ngram Frequency (CNF; log-transformed)
what_DDQ	7.4911	what_DDQ	7.643962
what_DDQ ‘s_VBZ	3.2205	what_DDQ ‘s_VBZ	5.411646
’s_VBZ a_AT1	3.6639	what_DDQ ‘s_VBZ a_AT1	2.484907
a_AT1 mountain_NN1	10.106	what_DDQ ‘s_VBZ a_AT1 mountain_NN1	0.000000
mountain_NN1 for_IF	4.0000	what_DDQ ‘s_VBZ a_AT1 mountain_NN1 for_IF	0.000000
for_IF you_PPY	4.5734	what_DDQ ‘s_VBZ a_AT1 mountain_NN1 for_IF you_PPY	0.000000

Another frequency-based measure used here is the *number of once-attested ngrams* per TCU (*N_0_CNF*). This novel measure is based on the following rationale.

As noted, listeners seek to predict the TCU’s lexico-syntactic path in order to anticipate how and when the TCU is going to end (cf. Magyari et al., 2014: 2537; cf. also De Ruiter et al., 2006). While, clearly, successful anticipation and hence response speed may depend on a number of factors, such as syntactic affordances (Barthel and Sauppe, 2019) and early or late placement of key information (Bögels et al., 2015), a likely additional factor is the extent to which an unfolding utterance aligns with pre-established phraseological usage that members of a language community have accumulated and stored through their experience as language users (DeLong et al., 2005: Hoey, 2005). Based on this resource, they will more easily predict the trajectory of common word combinations than that of unusual or even novel combinations they have never experienced before (e.g., Corps et al., 2018; Magyari et al., 2014, p. 2537).

The variable recording the number of only once-attested ngrams per TCU, *N_0_CNF,* aims to capture the moment when the TCU-so-far has left behind the ‘trodden paths’ of everyday usage and presents the listener with a sequence of words that is, beyond this one occurrence, not yet attested— at least not in the corpus. We refer to this moment as the 0-point (as the logarithm of 1 is 0). To the extent that a corpus can be seen as a microcosm reflecting the macrocosm of a language (cf. Section 5), that 0-point would demarcate the entry point into uncharted phraseological territory: a stringing together of words that has no precedent in a language user’s experience. Listeners, lacking that experience, have no blueprint to rely on, and predicting the TCU’s lexico-syntactic path from that point onwards likely becomes a challenging task.

To illustrate, consider Table 5, which, for the example question *What’s a mountain for you?* gives the number of only once-attested ngrams, *N_0_CNF,* and Cumulative Ngram Frequencies (CNF) representing the total log-transformed frequencies of each ngram (1-gram, 2-gram, 3-gram, 4-gram, etc.) in the TCU. The log-transformed CNF values for *What’s a mountain for you?* already on *mountain* hit the floor, that is, the minimum value 0, indicating that the ngram token *what_DDQ ‘s_VBZ a_AT1 mountain_NN1* occurs just once in the corpus. Inevitably, the subsequent 4-gram *what_DDQ ‘s_VBZ a_AT1 mountain_NN1 for_IF* and the 5-gram *what_DDQ ‘s_VBZ a_AT1 mountain_NN1 for_IF you_PPY* also occur just once in the corpus. Thus, the total number of only once-attested ngrams for which there is no prior attestation in the listener’s language experience, in this example, is 3.

**Figure 2:**
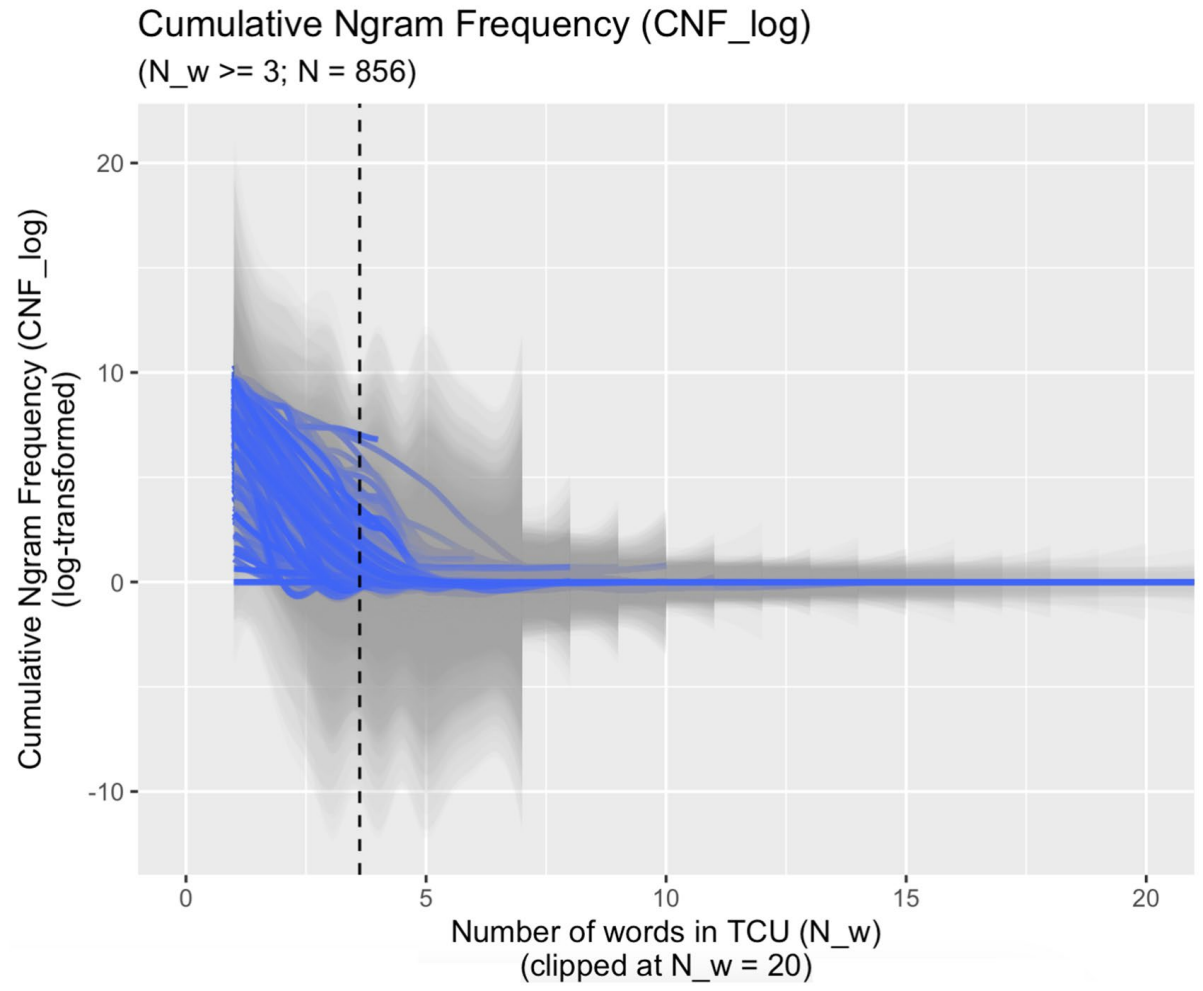
Quintic slope of word frequencies in TCUs (three-word minimum length) in the question and storytelling subsets; *position_rel*: relative positions of words in the TCU (0–1); *F_norm_log*: log-transformed normalized frequencies.

As shown in Figure 2, in the 856 TCUs on which model #2 is based, the first ngram in each TCU that is attested only once (and, hence, has CNF_log = 0) occurs early on: the average word position of once-attested ngrams is 3.62. Note, however, that this average reflects the 733 TCUs (out of 856) in which the 0-point *is* reached; in 123 TCUs, all ngrams are attested more than once and the 0-point is never reached.

The measure for the number of only once-attested ngrams, *N_0_CNF,* is exploratory in character, and we feel justified to use it in the analyses, considering that, essentially, how conversationalists use word frequencies in conversation and what role frequencies play, if any, in turn transition is still largely *terra incognita*.

#### 2.5.7 Statistical analysis

RQ #1—*Do word frequencies in TCUs follow an S-shaped pattern?*—was addressed using a mixed-effects model. To handle the variance in lengths of the TCUs (as measured in terms of number of words), a relative positional measure *position_rel was* computed for each TCU, assigning as many equi-distanced values between 0 and 1 as there were words in the TCU (e.g., the relative positions of the five words in a 5-word TCU are 0, 0.25, 0.5, 0.75, and 1). The fixed effects in the model were the log-transformed normalized frequencies *(F_norm_log)* (as the dependent variable) and *position_rel* (the independent variable); file/participant was modeled as a nested random factor. To account for (the expected) non-linear effects of relative position within the TCU (*position_rel*), we modeled this predictor using orthogonal polynomial terms. Models including polynomial terms of increasing order (from 1st to 6th) were fit successively. Model comparisons were conducted using AIC, BIC, and likelihood ratio tests to determine the appropriate degree of polynomial to retain. We restricted the analysis to TCUs with at least three words. This ensured that the trajectory of word frequencies could, in principle, display the hypothesized three-step pattern. Model comparisons (AIC/BIC) further indicated improved fit when two-word TCUs were excluded.

To address RQ #2—*Which frequency-related measures predict that a TCU will be followed by turn transition or continuation?*—a generalized mixed-effects logistic regression model was fitted to the data, with *Turn Transition* (TT) as the binary outcome variable. The predictor variables were:

- *S_DiffSecndFirstHalf*: The difference of the mean *surprisal* in the second half of the TCU minus the mean of *surprisal* in the first half. This conceptualization of *surprisal* is based on Trujillo and Holler’s (2024) finding that, in English conversation, *surprisal* in a turn’s second half is greater than in the first half.
- *F_DropLastThird*: The difference of the largest word frequency in the first two-thirds of a TCU minus the smallest word frequency in the last third of the TCU. This conceptualization of word frequency builds directly on the assumption that the drop at turn/TCU endings might be used as a turn completion cue.
- *N_0_CNF*: The number of once-attested ngrams in the TCU. As noted, the assumption here is that the listener’s task of predicting the trajectory and, finally, the end point of the TCU is becoming challenging once the speaker’s talk arrives at, and extends beyond, the first 0-point (the first only once-attested ngram). How that challenge impacts the anticipation of turn completion is yet an open question.

The random variable was *FileSpeakerID*, a combination of participant and recording ID.

In the remainder of this article, we will describe, in Section 3, the results of our enquiries into our two research questions, and then, in Section 4, discuss these results, before we conclude the study in Section 5.

## 3 Results

### 3.1 RQ#1 - do word frequencies in TCUs follow an S-shaped pattern?

Our mixed-effects model predicts log-transformed normalized word frequency (*F_norm_log*) based on a fifth-degree polynomial of relative position in the turn (*position_rel*), with random intercepts for individuals (*Person_anon*) nested within files (*File*). Model comparison using AIC/BIC and likelihood ratio tests indicated that including up to the fifth-order polynomial significantly improved model fit over lower-order models, while including the sixth-order polynomial did not. The model confirms that word frequency follows a complex non-linear pattern across turn positions, which seems to align with the S-shaped effect reported in prior research.

The model summary is given in Table 6.

**Table 6:** Model summary for Model RQ#1; Formula: *F_norm_log ~ poly(position_rel, 5) + (1 | File/Person_anon)*.

Random effects			
Groups	Name	Variance	Std. Dev.
Person_anon: File	(Intercept)	0.01351	0.1162
File	(Intercept)	0.01153	0.1074
Residual		5.11856	2.2624
Number of obs: 6824, groups: Person_anon: File, 44; File, 16			

Fixed effects					
	*β*	Std. Error	df t value	Pr(>|t|)	*p*-value
(Intercept)	0.53032	0.04499	13.479701	1.789	1.76e-08 ***
position_rel^1^	−74.52082	2.27249	6789.94250	−32.793	< 2e-16 ***
position_rel^2^	−7.12678	2.27354	6806.90308	−3.135	0.00173 **
position_rel^3^	−14.39053	2.26841	6789.61424	−6.344	2.38e-10 ***
position_rel^4^	−10.83934	2.26781	6816.12194	−4.780	1.79e-06 ***
position_rel^5^	−6.59510	2.26469	6789.67182	−2.912	0.00360 **

The Random Effects suggest that there is some variability in word frequency across different individuals within files (Variance = 0.01351, SD = 0.1162) and that differences in files contribute to variability in word frequency (Variance = 0.01153, SD = 0.1074); the largest source of variation is residual (unexplained) variation, suggesting that factors other than position in the TCU may also influence word frequency (5.15657, SD = 2.2708).

Regarding the Fixed Effects, all polynomial terms up to the fifth order were statistically significant, providing strong evidence that the relationship between relative word position and normalized word frequency is highly non-linear. Although the large negative coefficient for the first-degree term reflects a strong overall downward trend from the beginning to the end of the TCU, the additional higher-order terms (quadratic through quintic) reveal systematic departures from this monotonic decline. Since the model employs orthogonal polynomials, the individual coefficients are not directly interpretable in terms of slope or curvature. Instead, their joint significance demonstrates that the trajectory of word frequency across positions contains multiple inflection points. To judge by the curve depicted in Figure 2, these inflection points are largely consistent with an S-shaped distribution reported in previous research, which could indicate an initial drop, a plateau, and then a sharp final drop.

**Figure 3:**
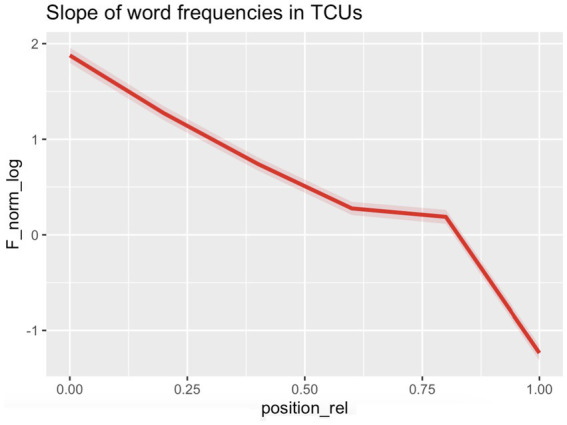
Cumulative Ngram Frequency (*CNF_log*) in the data used for model #2 (addressing RQ #2); dotted line: mean word position of once-attested ngram in TCU (mean = 3.62).

### 3.2 RQ #2 - which frequency-related measures predict that a TCU will be followed by a turn transition or continuation?

The logistic fixed-effects model, model #2, to address RQ #2 builds on the back of the results of the model to address RQ #1. While model #1 confirms the S-shape pattern for TCUs, including specifically the steep drop at TCU ends, model #2 takes as its starting point that steep drop in frequency and operationalizes it as *F_DropLastThird* as one predictor beside the difference of the mean *surprisal* in the second half of the TCU minus the mean of *surprisal* in the first half, *S_DiffSecndFirstHalf,* and the number of only once-attested ngrams, *N_0_CNF*.

The model included *FileSpeakerID* as a random intercept to account for variability across speakers and files. However, the estimated variance for this effect was notably large (70.09), suggesting it might not be essential for explaining variation in turn transitions. To assess whether *FileSpeakerID* significantly improved model fit, we compared the revised model with a reduced model excluding this random effect using a likelihood ratio test (LRT). The model comparison revealed that removing *FileSpeakerID* resulted in a significantly poorer fit (χ^2^ = 601.26, df = 1, *p* < 0.001), justifying its inclusion in the model.

The summary of the model is given in Table 7; the reference level for turn transition (*TT*) is TT = “yes”:

**Table 7:** Model summary RQ#2; *TT ~ S_DiffSecndFirstHalf + N_0_CNF + F_DropLastThird + (1 | FileSpeakerID).*

Random effects			
Groups	Name	Variance	Std. Dev.
FileSpeakerID	(Intercept)	70.09	8.372
Number of obs: 856, groups: FileSpeakerID, 44			

Fixed effects				
	*β*	Std. Error	*z* value	Pr(>|z|)
(Intercept)	−9.483750	1.604535	−5.911	3.41e-09 ***
S_DiffSecndFirstHalf	0.008203	0.046911	0.175	0.861
N_0_CNF	0.024144	0.035064	0.689	0.491
F_DropLastThird	0.063434	0.012198	5.200	1.99e-07 ***

Among the three predictors, the difference of the mean *surprisal* in the second half of the TCU minus the mean of *surprisal* in the first half, *S_DiffSecndFirstHalf,* (*β* = 0.008203, *p* > 0.5), and the number of only once-attested ngrams in the TCU, *N_0_CNF,* (β = 0.024144, p > 0.5) do not have a significant effect. The only significant predictor of *Turn Transition* (TT) is *F_DropLastThird* (β = 0.063434, p < 0.001). Its effect is positive, that is, increases in the frequency drop in the last third of the TCU are associated with increases in the log-odds that turn transition (in questions as opposed to stories) will occur.

## 4 Discussion

In this article, we explored the possibility that frequency and frequency-related measures serve as resources for the listener to (advance-)project (imminent) turn completion. We approached this possibility from two angles relating to two research questions.

**Figure 4:**
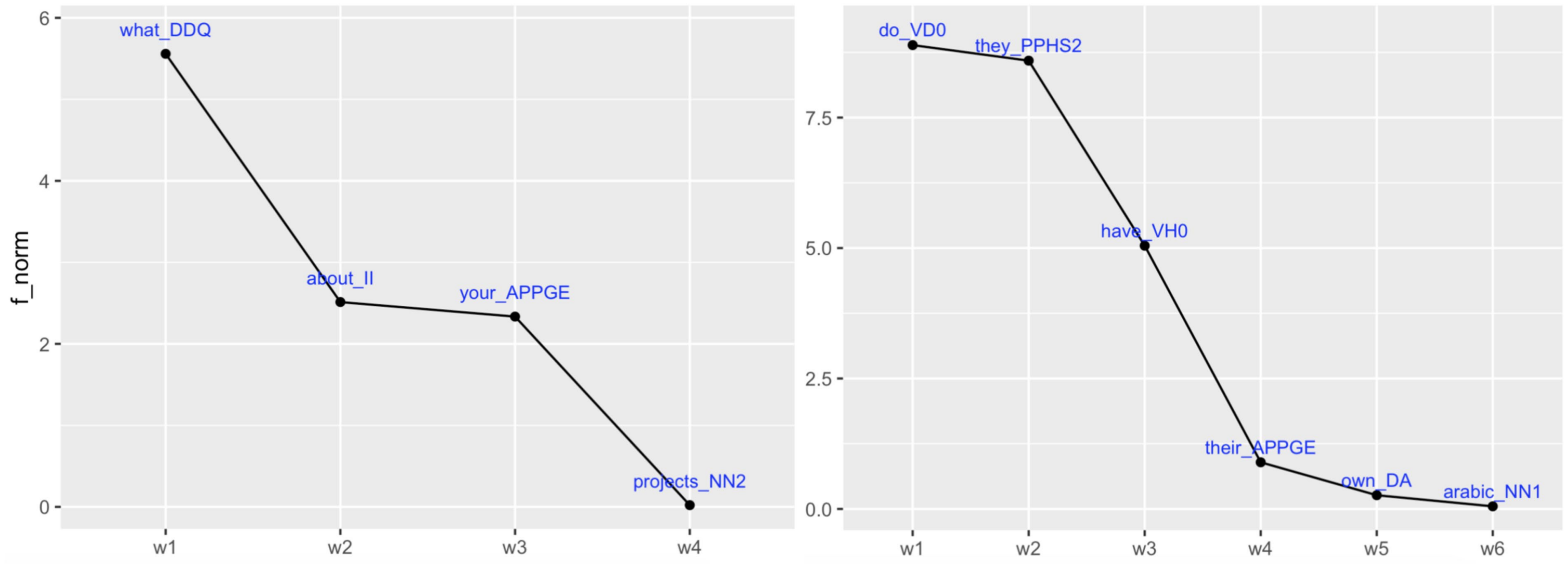
Examples of question TCUs with S-shaped word frequencies; *f_norm*: word frequencies in FreMIC normalized by 1,000.

Our first research question—*Do word frequencies in TCUs follow an S-shaped distribution?*—was answered in the positive: on analyzing the log-transformed normalized word frequencies in TCUs, we found an S-shaped distribution, exhibiting a drop in initial position(s), a more level stretch in mid-TCU position(s), and a sharp drop in final position(s). For illustration, consider Figure 4, showing the trajectories of word frequencies of two questions:

This finding is noteworthy with regard to previous findings of a similar S-shape of frequencies in two ways. First, the S-shape in the literature was found in much larger datasets: in Rühlemann and Barthel (2024), for example, the underlying data comprised almost 300,000 utterances from the conversational component of the British National Corpus (BNC); in the present study, the pattern emerged from only 974 units. This indicates the robust strength of the pattern. Second, the underlying units of observation in the literature were quite different. In Yu et al. (2016), for example, it was the (written) sentence (in data from the written component of the BNC); in Klafka and Yurovsky (2021) and in Rühlemann and Barthel (2024), it was utterances (bounded by speaker change and/or pauses) but not turns in any strict conversation-analytic sense; in the present study, the pattern was found in the smallest interactionally significant unit, the TCU. Given that the frequency of a word is negatively correlated with its information content (Yu et al., 2016; Rühlemann and Barthel, 2024), the S-shape distribution of frequencies in TCUs suggests that information content is climactically ordered not only in sentences or utterances but even in TCUs. For illustration, in the two TCUs in Figure 4, the informational peak is clearly on the last words, *projects* and *arabic*. Further, assuming that conversation represents the “core matrix for human social life” (Stivers et al., 2009) and the central context of language use from which others are departures (Goodwin and Heriage, 1990, p. 298), the finding points to the possibility that the informational asymmetry in sentences in writing may have formed in the mold of the TCU.

To address the second research question—*Which frequency-related measures predict that a TCU will be followed by turn transition or continuation?—*a logistic mixed-effects model was fitted with *Turn Transition* (*TT*) as the binary outcome variable. The model with the three factors suggested that neither *S_DiffSecndFirstHalf*, which captures *surprisal,* nor *N_0_CNF*, which captures the number of once-attested ngrams per TCU, discriminate significantly between turn-yielding in questions (TT = “yes”) and turn-holding in storytelling (TT = “no”). The only predictor that was found to have that discriminatory power was *F_DropLastThird:* the larger the drop in frequency in the last third of the TCU, the larger the log-odds that turn transition in questions will occur.

How to make sense of these findings? To reiterate, the findings were based on a juxtaposition of question-TCUs in QA sequences that did result in speaker change (TT = “yes”) and narrative TCUs in storytellings that did not lead to speaker change (TT = “no”). So all the findings, be they negative or positive, strictly relate to that action-transition nexus.

The *suprisal* variable *S_DiffSecndFirstHalf* and the phraseological variable *N_0_CNF* have in common that they represent resources listeners may deploy to predict the lexico-syntactic trajectory and anticipate the end point of the speaker’s talk (Magyari et al., 2014; De Ruiter et al., 2006). In the present study, these two variables fail to predict the turn transition in questions as opposed to stories. This failure does not invalidate these variables for future studies of turn transition. In different research scenarios, the variables may well be capable of discriminating turn-yielding TCUs from turn-holding ones.^10^ Particularly, the novel variable for only once-attested ngrams, *N_0_CNF*, is promising enough to be tested in future studies for its impact on listeners and their ability to predict a TCU’s lexico-syntactic course.

The main finding of the model is that the drop in frequency is sharper in turn-transitioning questions than in turn-holding story TCUs. This is intriguing and, at first sight, counterintuitive as storytelling epitomizes “displaced talk,” which may require extending the “discoursal horizon” beyond the here-and-now; that extension may necessitate a more diverse vocabulary (indicating time and place, giving characters’ names, describing story objects and characters’ actions) than asking an information-seeking question related to the immediate situational or sequential context. A greater diversity of the vocabulary inevitably entails less-frequent words. Tentatively, however, story TCUs and question TCUs might differ in how rarer words are distributed within the TCU: while in story TCUs, the rarer (and more informative) words might be distributed more uniformly, their distribution in question TCUs might be more asymmetrical, with greater weight toward the TCU end. This hypothesis is explored in a keyness analysis in the following section.

### 4.1 Follow-up analysis: key c7 tags in TCU intervals

Keyness analysis (Scott and Tribble, 2006) is a statistical method that identifies items of unusual frequency in a target corpus in comparison with a reference corpus. While in most analyses of keyness, the aim is to work out *words* that are key, we are going to apply the keynesss method to the c7 PoS tags. The aim is to test the hypothesis that the distribution of rarer word classes in question TCUs is more asymmetrical, with greater weight toward the TCU end, than in story TCUs.

**Figure 5:**
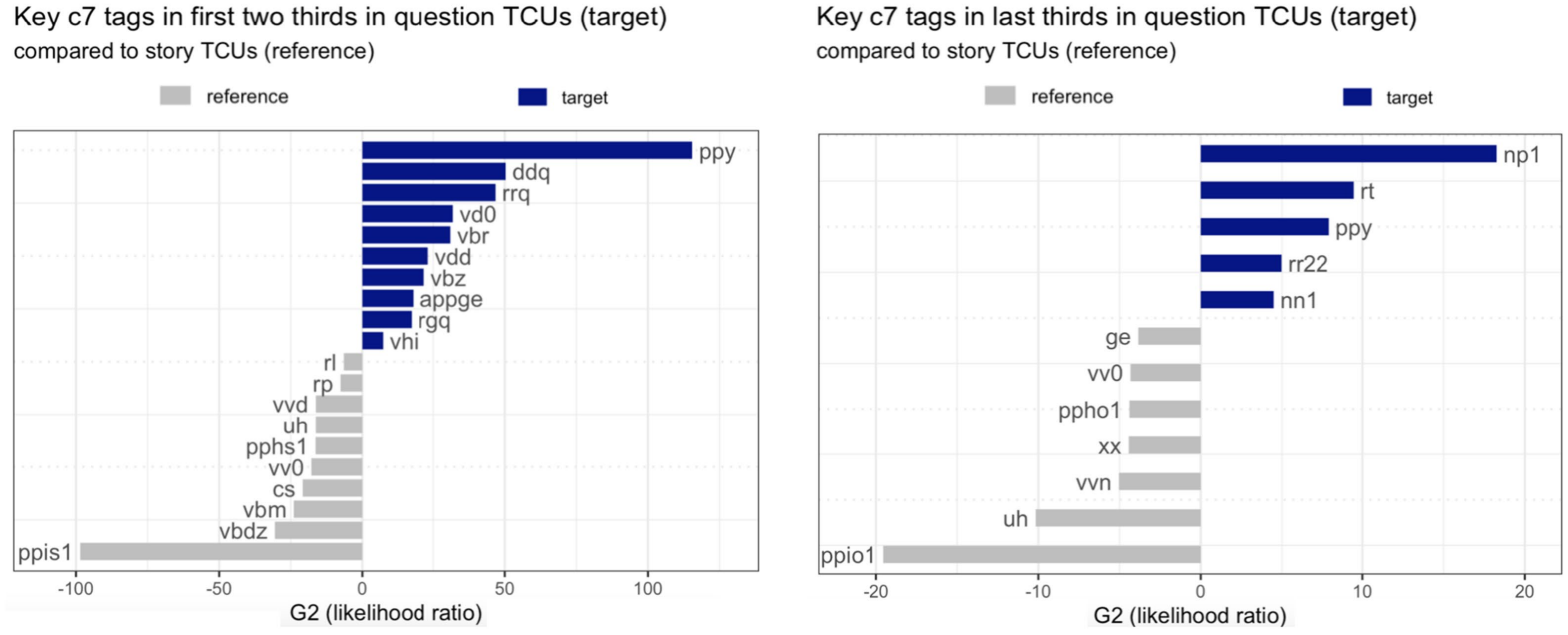
Top-most key c7 tags (with *p* < 0.05 and absolute log ratio > = 1) in different intervals in question TCUs (target corpus) compared to story TCUs (reference corpus): *left panel:* top 10 most key c7 tags in first two-thirds of question TCUs (blue bars) compared to first two-thirds of story TCUs (grey); *right panel:* all key c7 tags in last third of question TCUs (blue) compared to last third of story TCUs (grey).

To this end, word-tag combinations (e.g., *how_RGQ*) were stripped of the word part so that only the c7 tag remained (*RGQ*). Further, two subcorpora were compiled: one for the first two-thirds of TCUs, one for the last third of TCUs, in which model #2 above found a more pronounced drop in frequency for question TCUs than for story TCUs. Finally, using the R packages *quanteda* and *quanteda.textplots*, questions were defined as the target corpus and story TCUs as the reference corpus and *key* c7 tags in questions, as compared to stories, were computed using G^2^ (likelihood ratio), a measure of how strongly the observed frequency of a tag deviates from what would be expected by chance between the target and reference corpus. Also, log ratios were computed as an effect size measure (cf. Brezina, 2018). The top-most key c7 tags are shown in Figure 5.^11^

As shown in Figure 5, the most key c7 tags in the early intervals are PPY in questions and, respectively, PPIS1 in stories, with the former designating the second-person personal pronoun, *you* (the sixth most common word in FreMIC, cf. Table 3 above), and the latter, the first-person pronoun, *I* (by far the most common word in FreMIC; cf. Table 3 above). These are very strong but obvious differences, as most questions are addressed to the interlocutor(s) (e.g., *are you guys brothers?*) and many stories are first-person stories in which the storyteller is the main protagonist. The second most key c7 tags in the early intervals are DDQ in questions, i.e., *wh*-determiners, and VBDZ in stories, i.e., the past tense form *was*. These are also to be expected, as a large chunk of the questions are *wh*-questions, and most stories relate events that happened in the past (see also the key tag VVD for stories). What is notably *missing* from the early intervals, both in questions and stories (at least among the top 10 most key tags; s. Supplementary Materials 2 and 3 for the full lists of key tags), are tags for any type of nouns. This absence is noteworthy not only because nouns are by far the most type-rich category (cf., for example, the small inventory of pronouns) and by far the most hapax-rich category (hapax legomena are words that occur just once in a corpus and have hence the lowest possible frequency; cf. Rühlemann and Barthel, 2024). The absence is also noteworthy because nouns “carry most of the lexical content, in the sense of being able to make reference outside language” (Stubbs, 2001, p. 40; Biber et al., 1999, p. 232), and their use is “felicitous only in contexts of information novelty, disambiguation needs, or topic and perspective shifts” (Seifart et al., 2018, p. 5721). So, nouns do not play a key role in the early intervals, either in question TCUs and story TCUs. Where nouns *do* come in is in the last interval—but only in question TCUs, not in story TCUs (see the full key tag lists in Supplementary Materials 2 and 3). In the last third in question TCUs, by far the most key tag is NP1 (for singular proper noun), and the fifth most key tag is NN1 (for singular common noun). In the late interval in story TCUs, by contrast, it is the c7 tag UH, that is, interjections (often at the beginning of direct speech), VVN, that is, the past participle of lexical verbs, and VV0, that is, the base form of lexical verbs, that are key. Here, now lies the explanation to the result of model #2, which indicated that the frequency drop is more pronounced in question TCUs than in story TCUs: the drop in frequency is sharper as nouns, the most informative and potentially rarest type of word, are more asymmetrically distributed toward the TCU end in question TCUs than in story TCUs.

Table 8 shows for each social action type, four TCU examples that are “prototypical” in the sense that they include words with key c7 tags for the first two-thirds and, respectively, the last third.

**Table 8:** Example TCUs with key c7 tags; emboldened items represent the w_c7 tag that had the highest frequency in the early intervals (F_max) and, respectively, the w_c7 tag that had the lowest frequency in the late interval (F_min); F_Drop *(F_DropLastThird)* is calculated from the difference of F_max and F_min.

Type	Early intervals (first two thirds)	Late interval (last third)	F_max	F_min	F_Drop
question	do_VD0 **you_PPY** guys_NN2 need_VV0 to_TO go_VVI back_RP	**ikea_NP1** anytime_NNT1 soon_RR	22.92	0.03	22.89
question	did_VDD you_PPY get_VVI **the_AT**	**poem_NN1** email_NN1	25.82	0.01	25.82
question	**you_PPY** ever_RR played_VVD like_II	a_AT1 **banjo_NN1**	22.92	0.02	22.90
question	did_VDD **you_PPY** get_VVI anything_PN1 out_II21 of_II22	that_DD1 **relationship_NN1**	22.92	0.10	22.82
story	**and_CC** he_PPHS1 immediately_RR the_AT second_NNT1 we_PPIS2 got_VVD on_II	just_RR **zoned_VVN** in_II us_PPIO2	26.90	0.01	26.89
story	**i_PPIS1** do_VD0 n’t_XX think_VVI	they_PPHS2 **care_VV0**	43.78	0.02	43.76
story	she_PPHS1 said:VVD oh_UH **i_PPIS1** was_VBDZ	**invited_VVN** too_RR	43.78	0.04	43.74
story	uh_UH **and_CC** his_APPGE position_NN1 as_II a_AT1	diplomat_NN1 is_VBZ **cut_VVN**	26.90	0.01	26.89

Is the TCU-final frequency drop a *turn*-completion cue, regardless of social action type? This question cannot definitively be answered by this study, which compared turn-final question TCUs with turn-medial story TCUs. A *general* turn-completion signaling function for frequency is, however, unlikely. For it would presuppose that speakers manipulate frequencies depending on whether they wish to yield or keep the turn. A manipulation of frequencies could only be achieved if the speaker were skilled enough to use one way of phrasing for one purpose and another way of phrasing for the other purpose. That certainly overestimates a speaker’s conscious control over what they say and their stylistic versatility, and it underestimates the constraints imposed by constituent order, which is strict in English, leaving little room for *in situ* variation. It appears more plausible that the frequency drop observed in this study, both in response to RQ #1 and RQ #2, functions as a *TCU* completion cue. Whether that TCU is (intended by the speaker) as the turn-final one is likely signaled by other, far less rules-governed *prosodic* cues such as turn-final lengthening (Duncan, 1972; Local and Walker, 2012; Bögels and Torreira, 2015), creaky voice (Ogden, 2001; Redi and Shattuck-Hufnagel, 2001), audible outbreath (Local and Walker, 2012; Torreira et al., 2015), and pitch drop (Beattie et al., 1982; Duncan, 1972; Bögels and Torreira, 2015). On this view, turn-completion is most likely signaled by the speaker and processed by the listener in *multimodal clusters*, in which the TCU-final drop in word frequency is one of the several components.

## 5 Conclusion

FreMIC is a small corpus. Its smallness suggests that the findings should be treated with caution. For example, normalized frequencies may not yet be completely stable, and the speed with which, in the present data, cumulative ngrams become attested only once—on average, on the fourth word—may be exaggerated in FreMIC compared to larger corpora, where multi-word combinations that occur just once in FreMIC have a higher chance of occurring more frequently. In larger corpora, TCUs will likely reach that juncture at a later point.

The present findings hold for English conversation. To what extent they can be generalized to more languages is an open question. The generalizability may already prove difficult with closely related SVO languages such as, for example, German, which may be among the “front-loaded information languages” (Trujillo and Holler, 2024), in which the first half of utterances is information-heavier than the second half (unlike in English, which is “back-loaded,” meaning the informational peak occurs in the second half of utterances) In the relatively few languages of the world where the basic constituent order does not start with the subject constituent (c. 17% of all languages; cf. Hammarström, 2016), such as Jarawa (spoken on the Andaman Islands, India; OSV), the distribution of frequencies and related measures across words in turns will likely diverge substantially from that in English conversation (where the subject is typically a high-frequency pronominal form; cf. Rühlemann and Barthel, 2024), and it is doubtful whether in these languages any similar TCU-final frequency drop can be observed. This, however, is not to suggest that frequency patterns in these languages can never play any role in signaling that the current speaker is about to stop speaking and ready to hand over to another participant. The patterns, if any, might simply be of a different kind (for example, in an OVS language, a TCU-final *rise* in frequency might be construed by listeners as a cue that the speaker is done).^12^

Finally, frequency and frequency-related measures cannot in themselves fully explain turn completion or continuation. Frequency measures will no doubt enter into important interactions with other turn-completion cues (Bögels and Torreira, 2015, p. 55) and/or form multimodal packages. Future studies should therefore exhaustively incorporate the diverse set of turn-completion cues not only on the lexical/verbal level but also on the gestural/visual and prosodic/vocal levels. Only thus will it be possible to gain a *comprehensive* view of how speakers give the green light to their interlocutors that they are done and that someone else can now speak.

These limitations notwithstanding, this study does suggest that, in English conversation, word frequencies form an S-shaped pattern in TCUs (RQ #1) and they do discriminate turn-final question TCUs and turn-medial storytelling TCUs (RQ #2). Information extracted from word frequencies may hence serve listeners in conversation as cues to anticipate turn completion in questions as opposed to turn continuation in stories. Whether that information also discriminates other types of social action remains to be investigated in future research.

## Data Availability

The datasets presented in this study can be found in online repositories. The names of the repository/repositories and accession number(s) can be found at: The data and the R code are openly available in Open Science Framework at https://osf.io/ygnze/.

## Footnotes

^1^The schematic representation only depicts turn-final go-signals; it does not depict advance-projecting turn completion cues, whose onset may be much earlier in the turn.

^2^Long-distance projection appears to play a smaller role in estimating turn endings than one-off final cues. Corps et al. (2018) found that while content predictability enables listeners to prepare a response early, it does not guide them in deciding when to begin articulating it. Likewise, Bögels and Torreira (2015) demonstrated that prosodic features in the final word—but not in earlier ones—shaped turn-end judgments, indicating that final cues carry more weight than long-range anticipation.

^3^ http://ucrel-api.lancaster.ac.uk/claws/free.html

^4^Another advantage is that the underlying grammatical words in contracted forms are recognized and tagged separately; e.g., *gonna* is tagged *gon_VVGK na_TO.*

^5^Identifying such QA sequences is anything but trivial. Questions may remain unanswered, involve a *wh*-pronoun but do not seek information but affirmation of the stance displayed in the question (as in rhetorical questions); or they may get responded to but not in a type-fitted manner by the selected nex-speaker but by a third, non-selected party (Lerner, 2019) who inserts some (intrusive) talk that does not provide the sought information. Another complicating factor is the occurrence of questions in turbulent turn-taking, for example due to multiple overlap, which make identification of question and particularly answer difficult.

^6^The steps involved were: (i) map the first segment (for example, *so wait*, in extract (11.c)) to the (full) IPU of which it is a part thereby also mapping it to the *c7* word-tag string associated with the full IPU; this mapping utilizes the fact that both units start at the same time in the recording and therefore have the same starting time in ELAN; (ii) convert CA transcription in TCU segments into orthographic transcription by removing all CA-related characters, comments, pauses etc. making use of regular expression; (iii) collapse all orthographic TCU segments into a single string; (iv) devise a function to map *c7* word-tags in the IPU to the matching orthographic TCU segments; (v) apply the mapping function.

^7^FreMIC is a small corpus, with less than 400,000 word tokens. This smallness may be seen as compromising its ability to reflect the macrocosm of *la langue*. However, the normalized frequencies obtained for the question *what’s a mountain for you?* from FreMIC shown in Table 4. roughly follow the same trajectory as the normalized frequencies for the same question obtained from the much larger conversational subcorpus of the British National Corpus, which comprises 4.2 million word tokens, where the frequencies are, in the order of the words in the question: 9.09, 25.4,18.3,0.025, 5.43, and 31.9.

^8^Note that the frequencies in this example do not neatly follow the S-shaped distribution that will be demonstrated in Section 3. The example is hence representative of (the many other) cases in the question and story samples that do not behave prototypically. Examples of TCUs in which the frequencies are more closely aligned with the S-shape will be given in Section 4.

^9^*Surprisal* on the TCU-first word, for which there is no prior word(s), is obtained from the negative log of the word’s frequency in FreMIC divided by the total number of words in FreMIC (cf. Rühlemann and Gries, 2020). An alternative method, which takes into account the fact that turn/TCU-first words are taken from a rather specialized portion of the vocabulary, may be more precise (cf. Rühlemann and Schweinberger, 2021). This method, however, could not be adapted to the present data (due to the unavailability of units identiied as *turns* in FreMIC).

^10^The number of once-attested ngrams (N_0_CNF) was found an important variable in a Random Forest analysis of *multimodal packages* discriminating between transition-ready question TCUs and transition-averse story TCUs; this analysis incorporated 14 predictors from the verbal, visual, and vocal modalities (Rühlemann, Auer, Gries, Holler, & Schulte, *In preparation*).

^11^The plot also includes the results for NN1 although the log ratio is <1 (*p* < 0.05); see Supplementary Materials 2 and 3

^12^I owe this idea to an anonymous reviewer.
