# Supplementary material for: Do frequency and frequency-related measures signal turn completion? An exploratory corpus study
Source: Front Psychol. 2025 Dec 4;16:1610179. doi: 10.3389/fpsyg.2025.1610179 (PMC12713358; doi:10.3389/fpsyg.2025.1610179)
Supplement: Supplementary file 1 [file Data_Sheet_1.pdf]

Supplementary material

Material 1: UCREL CLAWS-7 tag set

source: https://ucrel.lancs.ac.uk/claws7tags.html

APPGE	possessive pronoun, pre-nominal (e.g. my, your, our)
AT	article (e.g. the, no)
AT1	singular article (e.g. a, an, every)
BCL	before-clause marker (e.g. in order (that),in order (to))
CC	coordinating conjunction (e.g. and, or)
CCB	adversative coordinating conjunction (but)
CS	subordinating conjunction (e.g. if, because, unless, so, for)
CSA	as (as conjunction)
CSN	than (as conjunction)
CST	that (as conjunction)
CSW	whether (as conjunction)
DA	after-determiner or post-determiner capable of pronominal function (e.g. such, former, same)
DA1	singular after-determiner (e.g. little, much)
DA2	plural after-determiner (e.g. few, several, many)
DAR	comparative after-determiner (e.g. more, less, fewer)
DAT	superlative after-determiner (e.g. most, least, fewest)
DB	before determiner or pre-determiner capable of pronominal function (all, half)
DB2	plural before-determiner (both)
DD	determiner (capable of pronominal function) (e.g any, some)
DD1	singular determiner (e.g. this, that, another)
DD2	plural determiner (these,those)
DDQ	wh-determiner (which, what)
DDQGE	wh-determiner, genitive (whose)
DDQV	wh-ever determiner, (whichever, whatever)
EX	existential there
FO	formula
FU	unclassified word
FW	foreign word
GE	germanic genitive marker - (' or's)
IF	for (as preposition)

II	general preposition
IO	of (as preposition)
IW	with, without (as prepositions)
JJ	general adjective
JJR	general comparative adjective (e.g. older, better, stronger)
JJT	general superlative adjective (e.g. oldest, best, strongest)
JK	catenative adjective (able in be able to, willing in be willing to)
MC	cardinal number,neutral for number (two, three..)
MC1	singular cardinal number (one)
MC2	plural cardinal number (e.g. sixes, sevens)
MCGE	genitive cardinal number, neutral for number (two's, 100's)
MCMC	hyphenated number (40-50, 1770-1827)
MD	ordinal number (e.g. first, second, next, last)
MF	fraction,neutral for number (e.g. quarters, two-thirds)
ND1	singular noun of direction (e.g. north, southeast)
NN	common noun, neutral for number (e.g. sheep, cod, headquarters)
NN1	singular common noun (e.g. book, girl)
NN2	plural common noun (e.g. books, girls)
NNA	following noun of title (e.g. M.A.)
NNB	preceding noun of title (e.g. Mr., Prof.)
NNL1	singular locative noun (e.g. Island, Street)
NNL2	plural locative noun (e.g. Islands, Streets)
NNO	numeral noun, neutral for number (e.g. dozen, hundred)
NNO2	numeral noun, plural (e.g. hundreds, thousands)
NNT1	temporal noun, singular (e.g. day, week, year)
NNT2	temporal noun, plural (e.g. days, weeks, years)
NNU	unit of measurement, neutral for number (e.g. in, cc)
NNU1	singular unit of measurement (e.g. inch, centimetre)
NNU2	plural unit of measurement (e.g. ins., feet)
NP	proper noun, neutral for number (e.g. IBM, Andes)
NP1	singular proper noun (e.g. London, Jane, Frederick)
NP2	plural proper noun (e.g. Browns, Reagans, Koreas)
NPD1	singular weekday noun (e.g. Sunday)
NPD2	plural weekday noun (e.g. Sundays)
NPM1	singular month noun (e.g. October)
NPM2	plural month noun (e.g. Octobers)

PN	indefinite pronoun, neutral for number (none)
PN1	indefinite pronoun, singular (e.g. anyone, everything, nobody, one)
PNQO	objective wh-pronoun (whom)
PNQS	subjective wh-pronoun (who)
PNQV	wh-ever pronoun (whoever)
PNX1	reflexive indefinite pronoun (oneself)
PPGE	nominal possessive personal pronoun (e.g. mine, yours)
PPH1	3rd person sing. neuter personal pronoun (it)
PPHO1	3rd person sing. objective personal pronoun (him, her)
PPHO2	3rd person plural objective personal pronoun (them)
PPHS1	3rd person sing. subjective personal pronoun (he, she)
PPHS2	3rd person plural subjective personal pronoun (they)
PPIO1	1st person sing. objective personal pronoun (me)
PPIO2	1st person plural objective personal pronoun (us)
PPIS1	1st person sing. subjective personal pronoun (I)
PPIS2	1st person plural subjective personal pronoun (we)
PPX1	singular reflexive personal pronoun (e.g. yourself, itself)
PPX2	plural reflexive personal pronoun (e.g. yourselves, themselves)
PPY	2nd person personal pronoun (you)
RA	adverb, after nominal head (e.g. else, galore)
REX	adverb introducing appositional constructions (namely, e.g.)
RG	degree adverb (very, so, too)
RGQ	wh- degree adverb (how)
RGQV	wh-ever degree adverb (however)
RGR	comparative degree adverb (more, less)
RGT	superlative degree adverb (most, least)
RL	locative adverb (e.g. alongside, forward)
RP	prep. adverb, particle (e.g about, in)
RPK	prep. adv., catenative (about in be about to)
RR	general adverb
RRQ	wh- general adverb (where, when, why, how)
RRQV	wh-ever general adverb (wherever, whenever)
RRR	comparative general adverb (e.g. better, longer)
RRT	superlative general adverb (e.g. best, longest)
RT	quasi-nominal adverb of time (e.g. now, tomorrow)
TO	infinitive marker (to)

UH	interjection (e.g. oh, yes, um)
VB0	be, base form (finite i.e. imperative, subjunctive)
VBDR	were
VBDZ	was
VBG	being
VBI	be, infinitive (To be or not... It will be ..)
VBM	am
VBN	been
VBR	are
VBZ	is
VD0	do, base form (finite)
VDD	did
VDG	doing
VDI	do, infinitive (I may do... To do...)
VDN	done
VDZ	does
VH0	have, base form (finite)
VHD	had (past tense)
VHG	having
VHI	have, infinitive
VHN	had (past participle)
VHZ	has
VM	modal auxiliary (can, will, would, etc.)
VMK	modal catenative (ought, used)
VV0	base form of lexical verb (e.g. give, work)
VVD	past tense of lexical verb (e.g. gave, worked)
VVG	-ing participle of lexical verb (e.g. giving, working)
VVGK	-ing participle catenative (going in be going to)
VVI	infinitive (e.g. to give... It will work...)
VVN	past participle of lexical verb (e.g. given, worked)
VVNK	past participle catenative (e.g. bound in be bound to)
VVZ	-s form of lexical verb (e.g. gives, works)
XX	not, n't
ZZ1	singular letter of the alphabet (e.g. A,b)
ZZ2	plural letter of the alphabet (e.g. A's, b's)

Material 2: Key c7 tags with $p < 0.05$ and absolute log ratio >= 1 in early intervals (first two thirds) in question TCUs (target) compared to story TCUs (reference):

c7 tag	G2	p	n_target	n_reference	log_ratio
ppy	115.407637061465	0	208	52	2.06409509011958
ddq	50.2946927317434	1.3230527784458E-12	73	13	2.51922187701393
rrq	46.7817450357228	7.93487497929846E-12	61	9	2.76902402623669
vd0	31.8351952422361	1.67823984709159E-08	60	15	2.03910396122876
vbr	30.9743846045142	2.6145642317843E-08	61	16	1.97255742032182
vdd	23.0815450544426	1.55273926949473E-06	40	9	2.16635952378208
vbz	21.6839525408993	3.21469310782252E-06	106	53	1.06767966816573
appge	17.8559442480135	2.38273802513067E-05	42	13	1.72894046831527
rgq	17.3000330292857	3.19181304128113E-05	12	0	4.71829322411576
vhi	7.29159868237858	0.00692778012629047	13	3	2.0219696144469
vdz	4.69168795650391	0.0303088593480455	8	1	2.57693737487022
vhd	-4.07116522426235	0.0436211737146128	1	8	-2.42806330618815
rg	-4.4754480774651	0.0343851852059237	2	9	-1.85156238421519
rt	-5.91147582984004	0.0150425644740994	7	20	-1.37622437466853
rl	-6.48701358003414	0.0108665378438267	6	19	-1.51052546638012
rp	-7.62478601725594	0.00575715000894439	8	24	-1.45280996852383
vvd	-16.2389109487303	5.58354415522988E-05	20	56	-1.38818992345607
uh	-16.2472797230354	5.55893512229622E-05	11	40	-1.74185101248658
pphs1	-16.3413364348488	5.28974244701219E-05	17	51	-1.48278047589722
vv0	-17.8089257151999	2.44234827959655E-05	46	99	-1.02302877509458
cs	-20.8244601779221	5.03359775427814E-06	14	51	-1.75408249771461
vbm	-23.9511106655597	9.88132708656941E-07	1	23	-3.89518931661544
vbdz	-30.5490614444413	3.25529313505157E-08	24	81	-1.65958127577483
ppis1	-98.5140122300905	0	11	116	-3.26618715425623

Material 3: Key c7 tags with p < 0.05 and absolute log ratio >= 1 in late interval (last third) in question TCUs (target) compared to story TCUs (reference):*

c7 tag	G2	p	n_target	n_reference	log_ratio
np1	18.263949727415	1.92312400950634E-05	50	19	1.50807645075437
rt	9.47021217412797	0.00208835217330949	20	6	1.79237947334181
ppy	7.93459341378002	0.00484985234303748	20	7	1.58592859587439
rr22	4.97484652065337	0.0257184855218716	8	1	2.637767527394
nn1	4.48827953619581	0.0341280073340848	160	141	0.317038431155242
ge	-3.84335030260138	0.0499436306286588	1	8	-2.36723315366436
vv0	-4.32231213543284	0.037615778041502	8	20	-1.13482197650292
ppho1	-4.39088197661746	0.0361316175064408	5	15	-1.35949750488476
xx	-4.43410954661613	0.0352275912481502	0	6	-3.56517253127627
vvn	-5.03889669744334	0.0247842701508749	4	14	-1.55278880682044
uh	-10.1645208247575	0.00143169120744013	2	15	-2.49700102863469
ppio1	-19.5501063477845	9.79952813484619E-06	0	15	-4.81892912352205

* NN1 is included although log ratio < 1.
